# Clinical *Clostridium difficile*: Clonality and Pathogenicity Locus Diversity

**DOI:** 10.1371/journal.pone.0019993

**Published:** 2011-05-19

**Authors:** Kate E. Dingle, David Griffiths, Xavier Didelot, Jessica Evans, Alison Vaughan, Melina Kachrimanidou, Nicole Stoesser, Keith A. Jolley, Tanya Golubchik, Rosalind M. Harding, Tim E. Peto, Warren Fawley, A. Sarah Walker, Mark Wilcox, Derrick W. Crook

**Affiliations:** 1 Nuffield Department of Clinical Laboratory Sciences, Oxford University, John Radcliffe Hospital, Oxford, United Kingdom; 2 National Institute for Health Research, Oxford Biomedical Research Centre Programme, John Radcliffe Hospital, Oxford, United Kingdom; 3 Nuffield Department of Clinical Medicine, Oxford University, John Radcliffe Hospital, Oxford, United Kingdom; 4 Department of Statistics, University of Oxford, Oxford, United Kingdom; 5 Department of Zoology, Oxford University, Oxford, United Kingdom; 6 Department of Microbiology, The General Infirmary, Old Medical School, Leeds, United Kingdom; 7 Department of Microbiology, Institute of Molecular and Cellular Biology, University of Leeds, Leeds, United Kingdom; Institut de Pharmacologie et de Biologie Structurale, France

## Abstract

*Clostridium difficile* infection (CDI) is an important cause of mortality and morbidity in healthcare settings. The major virulence determinants are large clostridial toxins, toxin A (*tcdA*) and toxin B (*tcdB*), encoded within the pathogenicity locus (PaLoc). Isolates vary in pathogenicity from hypervirulent PCR-ribotypes 027 and 078 with high mortality, to benign non-toxigenic strains carried asymptomatically. The relative pathogenicity of most toxigenic genotypes is still unclear, but may be influenced by PaLoc genetic variant. This is the largest study of *C. difficile* molecular epidemiology performed to date, in which a representative collection of recent isolates (n = 1290) from patients with CDI in Oxfordshire, UK, was genotyped by multilocus sequence typing. The population structure was described using NeighborNet and ClonalFrame. Sequence variation within toxin B (*tcdB*) and its negative regulator (*tcdC*), was mapped onto the population structure. The 69 Sequence Types (ST) showed evidence for homologous recombination with an effect on genetic diversification four times lower than mutation. Five previously recognised genetic groups or clades persisted, designated 1 to 5, each having a strikingly congruent association with *tcdB* and *tcdC* variants. Hypervirulent ST-11 (078) was the only member of clade 5, which was divergent from the other four clades within the MLST loci. However, it was closely related to the other clades within the *tcdB* and *tcdC* loci. ST-11 (078) may represent a divergent formerly non-toxigenic strain that acquired the PaLoc (at least) by genetic recombination. This study focused on human clinical isolates collected from a single geographic location, to achieve a uniquely high density of sampling. It sets a baseline of MLST data for future comparative studies investigating genotype virulence potential (using clinical severity data for these isolates), possible reservoirs of human CDI, and the evolutionary origins of hypervirulent strains.

## Introduction

*Clostridium difficile* infection (CDI) is a major concern in healthcare settings worldwide. Symptoms range from mild diarrhoea to life threatening pseudomembranous colitis, with 6% mortality overall, rising to 13.5% in older patients [1]. Individuals may be asymptomatically colonised in the community, or acquire the bacteria nosocomially [2]. Risk factors predisposing colonised patients to develop symptoms include antibiotic treatment and advanced age [3]–[5].

The major *C. difficile* virulence factors are large clostridial toxins designated toxin A (TcdA) and toxin B (TcdB). TcdA and TcdB share 63% amino acid sequence similarity [6] and four functional domains; a N-terminal catalytic domain, an autocatalytic cysteine protease, a hydrophobic membrane translocation domain and a C-terminal receptor binding domain (RBD) [7], [8]. Evidence that TcdB alone is essential for virulence has been provided [9], however, more recent data indicate that both toxins are important [10]. TcdA and TcdB are encoded within the 19.6kb pathogenicity locus (PaLoc), together with three additional genes; *tcdC*, *tcdR* and *tcdE*. PaLoc gene expression is growth phase dependent. During early logarithmic growth, high levels of *tcdC* and low levels of *tcdA*, *tcdB*, and *tcdR* are transcribed; during stationary phase the converse is true [11]–[13]. It is therefore thought that TcdC and TcdR are negative and positive regulators, respectively, of toxin expression [11], [14].

The molecular epidemiology of *C. difficile* has been studied using many different genotyping methods [15]–[17]. This led to the identification of epidemic and hypervirulent genotypes associated with increased morbidity and mortality. One such strain emerged in 2000–2001 [18] causing large CDI outbreaks with high mortality [19]–[21]. This strain is designated BI by restriction endonuclease typing (REA), NAP1 by pulsed field gel electrophoresis (PFGE), ST-1 by multilocus sequence typing (MLST) and 027 by PCR-ribotyping [18], [19], [21], [22]. The production of toxin *in vitro* by PCR-ribotype 027 has been described as robust, but not significantly different to non-hypervirulent strains [23], and as 16 to 23-fold higher than non-epidemic strains [24]. In a human gut model that simulates CDI, the duration of cytotoxin production by PCR-ribotype 027 was markedly longer than that of PCR-ribotype 001 (23 versus 13 days), and was associated with increased prevalence of vegetative cells, but peak toxin titres were similar [25]. PCR-ribotype 027 also shows increased sporulation efficiency [23], [26].

PCR-ribotype 078 has also been described as hypervirulent since it can cause symptoms of similar severity to 027 [27]. This PCR-ribotype produces less TcdA and TcdB *in vitro* than 027, but more than other toxinotypes [28]. PCR-ribotype 078 is frequently isolated from livestock [28] and its incidence in human disease appears to be increasing [17], [29].

Two characteristics of the PCR-ribotype 027 PaLoc have been proposed to explain its virulence. Firstly, the 027-*tcdB*-RBD is genetically divergent from other strains, apparently conferring broader cell tropism and more rapid cell entry [30]–[32]. Secondly, the *tcdC* gene has a single nucleotide deletion causing a frameshift that truncates the protein. This has been postulated to remove log phase repression of toxin expression [18], [24], [33], [34]. PCR-ribotype 078 also encodes a truncated TcdC [34], caused by a single nucleotide substitution creating a stop codon. The precise frequency and distribution of these potential hypervirulence-promoting PaLoc variants within the *C. difficile* population structure is unclear. This is due to the lack of recent large scale studies assessing simultaneously the clinical *C. difficile* population structure, and the nucleotide sequences of PaLoc variants.

The *C. difficile* population structure is clonal [22], [35], [36], comprising five genetic groups or clades [22] which persist despite homologous recombination [37]. Existing data suggest a congruent relationship between *tcdC* variant and genotype [34], [36], [38], and possibly a similar relationship for TcdB-RBD, although these data are more limited [36]. Many genotypes, representing all five clades [22] are currently associated with CDI [17], [22], [29], [39], and data on their relative pathogenicity would assist patient management and infection control. In particular, the incidence of PCR-ribotype 027 has declined recently in many countries, and the virulence potential of the now endemic PCR-ribotype 027 relative to other endemic genotypes is less clear [5], [40].

Our aims were to define the TcdB-RBD and TcdC variants for 1290 recent clinical isolates collected from a large, contemporaneous cohort of consecutive CDI cases, and determine their relationship to the *C. difficile* population structure defined by MLST. This would facilitate study of the evolutionary mechanisms among *C. difficile* isolates representing a clearly defined collection of co-circulating strains, as well as the estimation of genotype pathogenic potential based on PaLoc *tcdB*-RBD and *tcd*C similarity to known hypervirulent genotypes. The size of the study and density of sampling in a single geographic location provides a baseline of *C. difficile* MLST data. *C. difficile* genotypes can vary with host species, geographic location and over time, [41]–[43]. Our data set, together with the inherent inter-laboratory comparability and portability of all MLST data, (http://pubmlst.org/cdifficile) will help facilitate comparative studies to understand the reservoirs of human CDI, its international transmission and the evolutionary origin of hypervirulent strains.

## Results

### *C. difficile* has a clonal population structure

A total of 69 STs were identified among the 1290 clinical isolates, 36 of which are described for the first time in this study. The relative abundance of the STs is summarised in Table 1, with additional details on the frequency of *tcdB*-RBD and *tcdC* alleles in Table S1. PCR-ribotype data representing each ST are presented in Table 1, Table S3 and Fig. S1 to contextualise the study. Eight additional STs were described previously (ST-20, ST-27, ST-29, ST-32, ST-38, ST-39, ST-40, ST-69) [22], and one (ST-30) was identified in a separate study of infants (data not shown). All 78 STs were included in the analysis of *C. difficile* population structure.

**Table 1:** Frequency of STs (n = 69) within the clinical isolate collection (n = 1290).

ST (n)	Clade	*tcdC*	Ribotype^1^	ST (n)	Clade	*tcdC*	Ribotype^1^
**1** (448)	2	Δ1stop	027	**48** (3)	1	WT	038, 104
**2** (86)	1	WT	020, 014, 076, 220	**56** (3)	1	WT	021
**8** (86)	1	WT	002	**72** (3)	1	WT	005
**42** (68)	1	WT	106, 174	**28** (2)	1	WT	020
**6** (59)	1	WT	005	**51** (2)	1	WT, Δ18	186, 249
**3** (54)	1	WT	001, 009, 072, 115, 262, 305	**57** (2)	1	WT	003
**44** (46)	1	WT	015	**75** (2)	1	WT	062
**5** (43)	3	TAAstop	023	**77** (2)	1	WT	011
**10** (43)	1	WT, Δ18	015	**19** (1)	1	WT	110
**14** (28)	1	WT	014	**21** (1)	1	WT	097
**11** (27)	5	TAAstop	078	**23** (1)	4	N/A	138
**9** (22)	1	WT, Δ18	081	**24** (1)	1	WT	202
**7** (20)	1	WT	026	**25** (1)	3	TAAstop	023
**37** (19)	4	WT	017	**26** (1)	1	N/A	140
**17** (18)	1	WT	018	**31** (1)	1	WT	323
**58** (18)	1	WT	056	**34** (1)	1	WT	056
**49** (17)	1	WT	014	**50** (1)	1	WT	014
**16** (14)	1	WT	050	**52** (1)	1	WT	139
**13** (12)	1	WT	129	**59** (1)	1	WT	316
**54** (12)	1	WT	012	**60** (1)	1	WT	336
**33** (11)	1	WT	216	**65** (1)	1	WT	224
**36** (11)	1	WT	011	**66** (1)	1	WT	022
**45** (11)	1	WT	013	**67** (1)	2	WT	019
**18** (10)	1	WT	050	**68** (1)	1	WT	020
**12** (8)	1	WT	003, 225	**70** (1)	1	WT	021
**55** (8)	1	WT	070	**71** (1)	1	WT	013
**35** (7)	1	WT	046	**73** (1)	1	WT	103
**53** (7)	1	WT	103	**74** (1)	1	WT	319
**15** (6)	1	N/A	070	**76** (1)	1	WT	103
**43** (6)	1	WT	054	**78** (1)	1	WT	013
**22** (5)	3	TAAstop	023	**89** (1)	1	WT	005
**63** (5)	1	WT	053	**90** (1)	1	WT	005
**46** (4)	1	WT	320	**91** (1)	1	WT	326
**4** (3)	1	WT	137	**92** (1)	1	WT	228
**41** (3)	2	Δ18, Δ1stop	106, 194, 321				

STs are ranked in descending order of abundance. The clade that each ST belongs to is indicated, followed by the associated *tcdC* allele variant(s) classified as WT (wild type), Δ18 (having an 18 nt deletion in the coiled coil domain), and Δ1stop or TAAstop to indicate truncated variants. N/A: not applicable as all isolates of this ST were non-toxigenic. A more detailed version of this table is also provided as Table S1, to show the frequency of the *tcdB*-RBD and *tcdC* alleles associated with each ST.

1 PCR-ribotypes found in association with each ST. An ST can have more than one ribotype, however, the converse is also true and this, together with the numbers of isolates that were PCR-ribotyped is shown in Table S2 and Fig. S1.

**Figure 1:**
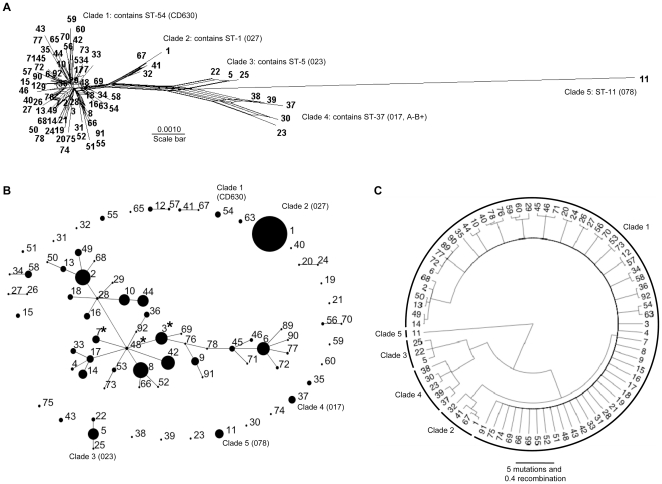
The *C. difficile* population is comprised of five clades with evidence of recombination within and among groups. (A) Phylogenetic network to illustrate relationships among 78 STs comprising the five clades. The networks were constructed using concatenated MLST loci and the program Neighbour-Net [44]. The clades containing well characterised isolates or genotypes, their ribotypes and STs are indicated (ST-89 was excluded as it differs from ST-6 by a single nucleotide deletion in the *sodA* locus only). (B) eBURST diagram to show the relationships among STs based on alleleic profiles [45]. Circle size for each of the 69 STs identified in the clinical isolate collection (total n = 1290) is proportional to the number of isolates. For completeness, one example of eight additional previously described STs (ST-20, ST-27, ST-29, ST-32, ST-38, ST-39, ST-40, ST-69) [22], and one (ST-30) identified in a separate study of infants were included to demonstrate their relationship to other members of the population. Well characterised representatives of each clade are indicated as in (A). STs identified in both toxigenic and non-toxigenic form are indicated by a star. (C) ClonalFrame analysis [46]. The five clades are indicated and branch lengths are measured in expected number of mutation and recombination events.

The total number of variable nucleotide sites was 127/3501 (3.6%), and amino acids 30/1167 (2.6%). The MLST loci were under strong conservative selection (dN/dS <1, Table S2) as expected for housekeeping genes. The sequences of each ST were concatenated and analysed using Neighbour-Net [44]. Five clades of closely related isolates were identified, representing deep branches of the phylogenetic tree (Fig. 1A). These clades were described previously [22], and although 36 new STs were identified in this study, they all fell within one of the five clades. The relative positions of the hypervirulent ST-1 (027) in clade 2 and ST-11 (078) in clade 5 are shown in Fig. 1A. Extensive networks were found in the ancestry of clade 1 (Fig. 1A), which suggest either homologous recombination or a lack of information to resolve these branchings. The relationships among the STs on the basis of allelic profile is shown by eBURST [45] (Fig. 1B). This analysis did not indicate the presence of many discrete clonal complexes not apparent by nucleotide sequence-based methods.

The five clades were also reconstructed by ClonalFrame [46], which accounts for the effect of recombination when reconstructing the genealogy (Fig. 1C). ClonalFrame was used to infer the numbers of point mutation and homologous recombination events in the *C. difficile* population. Recombination occurred approximately ten times less often than mutation (ρ/θ = 0.08 with credibility interval [0.04;0.13]), and introduced approximately four times fewer substitutions than mutation (r/m = 0.25 with credibility interval [0.12;0.42]).

### Detection of the Pathogenicity Locus

**Figure 2:**
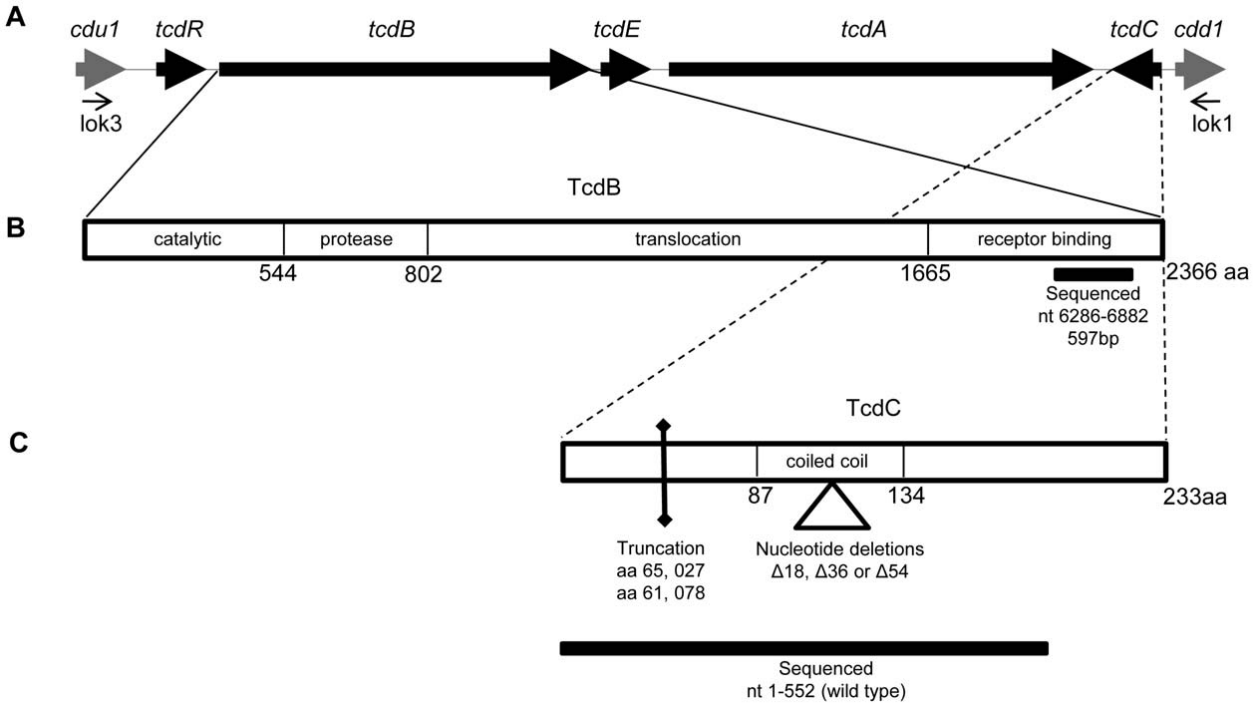
Relative positions of the *tcdB* and *tcdC* genes within the PaLoc and the sequences determined in this study. (A) Organisation of the five genes encoded by the 19.6 kb PaLoc of *C. difficile*, and two flanking genes containing the lok1/3 primer pair [47]. (B) The four functional domains of the 2366 amino acid TcdB protein, [7]. The region of the receptor binding domain (RBD) sequenced is indicated (horizontal black bar). The sequence determined is located within the C-terminal repetitive region which may bind to enteric cells *via* carbohydrate receptors [60]. (C) The 233 amino acid negative regulator TcdC. The location of the coiled-coil dimerization domain [14] is indicated; deletions found within the repetitive sequences of this domain are indicated by a triangle. The truncations found in PCR-ribotype 027 and 078 strains are indicated by a vertical bar. The sequence determined is indicated by a black horizontal bar.

The PaLoc was detected by PCR using oligonucleotide primers which amplify the *tcdB*-RBD and *tcdC* loci (Fig. 2A). Absence of the PaLoc was confirmed using the lok1/lok3 primer pair which bind chromosomal DNA either side of the ∼19.6kb PaLoc (Fig. 2A) [47]. The lok1/3 PCR amplifies 769 bp in the absence of the PaLoc, and thus identifies non-toxigenic isolates. A negative lok1/3 PCR in combination with positive *tcdB*-RBD and *tcdC* PCRs confirmed an isolate was toxigenic. A positive lok1/3 PCR and negative *tcdB*-RBD and *tcdC* PCRs indicated an isolate was non-toxigenic. All isolates conformed to either of these two patterns confirming that the PaLoc was present only in the previously described genomic location [47].

A total of 18 non-toxigenic isolates with seven STs were identified. Some STs had both toxigenic and non-toxigenic isolates (ST-3, ST-7 and ST-48), others were non-toxigenic only (ST-15, ST-23, ST-26, ST-30) (Table 1). Non-toxigenic strains appear not to represent a separate clade, since five were distributed throughout clade 1, (ST-3, ST-7, ST-48, ST-15, and ST-26) and two occurred in clade 4 (ST-23, ST-30) (Fig. 1).

### Genetic variation within the *tcdB*-RBD

**Figure 3:**
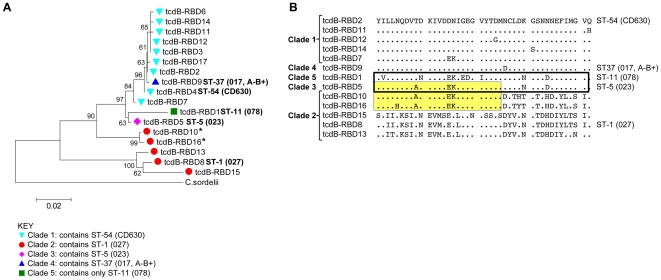
The association of *tcdB*-RBD alleles and clade is congruent. (A) Neighbour joining tree showing the relationships among the 17 *tcdB* allele sequences (597nt) and *C. sordelii* constructed using MEGA with bootstraps calculated using 1000 replicates. Coloured shapes indicate the clade (Fig. 1) with which each *tcdB* allele is associated. The number of variable nucleotide sites (excluding *C. sordelii*) was 97/597 (16.2%) and amino acids 42/199 (21.1%). * Indicates putative recombinants derived from clade 2 and clade 3 sequences. *C. sordelii* was used to root the tree as it encodes the closest known relative to *C. difficile tcdB*. (B) Alignment of the *tcdB*-RBD allele variable amino acid sites, relative to the sequence of the CD630 genome strain [51] (clade 1, allele *tcdB*-2). Alleles *tcdB*-3, *tcdB*-4, *tcdB*-6, and *tcdB*-17 are not shown as they have an amino acid sequence identical to *tcdB*-2. The bold box indicates the closely related sequences of clade 3 and clade 5; the yellow shaded box indicates putative clade 2/clade 3 *tcdB* recombinants; * in (A).

The PaLoc position of the *tcdB* gene, its functional domains and the region sequenced are summarised in Fig. 2A and B. *tcdB*-RBD sequences (597nt) were determined for all isolates. A total of 17 different alleles were identified. Each was assigned a number in the order of discovery and the sequences made available at http://pubmlst.org/cdifficile. The association of *tcdB*-RBD alleles and clades was congruent (Fig. 3A).

Clade 1 was most heterogeneous in terms of the number of STs (n = 65) (Fig. 1), but it was relatively homogenous within the *tcdB*-RBD, with nine alleles sharing 98.2% nucleotide and 97.5% amino acid identity (Fig. 3A and B). Clades 3, 4 and 5 each had a single clade-specific *tcdB*-RBD allele. The clade 4 *tcdB*-RBD9, was located among the clade 1 variants (Fig. 3A), differing by a single amino acid from its closest relative (Fig. 3B), an observation suggestive of recombination. Clades 3 and 5 *tcdB*-RBDs were closely related, sharing 97.7% nucleotide and 97.0% amino acid identity (Fig. 3A and B).

Clade 2 (containing ST-1 [027]) was most heterogeneous in terms of its *tcdB*-RBD alleles (Fig. 3A), the five *tcdB*-RBD alleles occurring in various combinations with four STs (Table 1); ST-1 (027), ST-41, ST-67, and one ST published previously ST-32 [22]. All ST-1 (027) isolates (n = 448) contained the expected divergent *tcdB*-RBD8 sequence [30], [31], which clusters with clade 2-associated *tcdB*-RBD13 and *tcdB*-RBD15 (Fig. 3A). Two clade 2 alleles (*tcdB*-RBD10 and 16) were located on a separate branch of the neighbour joining tree (Fig. 3A). They appear to have a complex admixed ancestry, with some polymorphism typical of clade 2 and clade 3 *tcdB*-RBD sequences, as well as a number of polymorphisms unique to these two alleles (Fig. 3B).

### Genetic variation within the *tcdC* negative regulator

**Figure 4:**
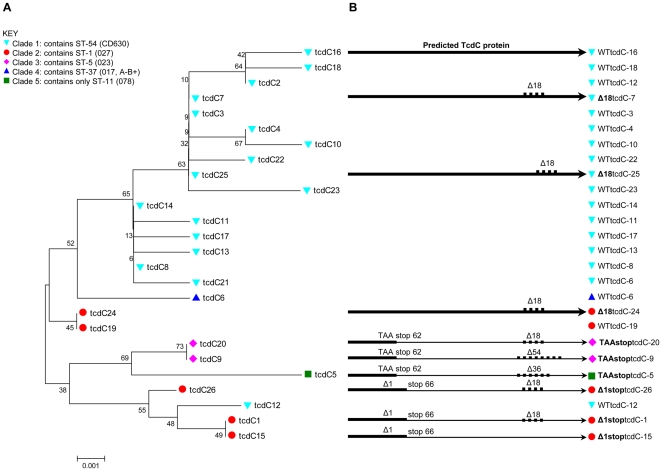
The association of *tcdC* alleles and clade is almost congruent. (A) Neighbour joining tree showing the relationship among *tcdC* variants and the clades. The variants were manually aligned in BioEdit and a neighbour joining tree was computed using MEGA with bootstraps calculated using 1000 replicates. Coloured shapes indicate the clade (Fig. 1) with which the *tcdC* allele was associated. Alleles appear the same if they differ only in terms of their relative deletion lengths which are indicated in (B). Alleles **WT**tcdC-8 and **WT**tcdC-14 appear the same on the tree as they differ by a single nucleotide at position 117, which is deleted in some other alleles and therefore contained an N in some variants in the alignment. (B) Predicted translation products from *tcdC* variants. Three different TcdC variants were found (i) wild type (**WT**) (ii) truncated by Δ1 at nucleotide 117 creating a frameshift and a stop codon at amino acid 66, (**Δ1stop**), or by a CAA to TAA substitution creating a stop codon at amino acid 62, (**TAAstop**) (iii) 18 nucleotides deleted (Δ18) within the coiled-coil dimerization domain. Further deletions within the untranslated sequence of clade 3 and clade 5 were 36 nucleotides long (Δ36) or 54 nucleotides long (Δ54). Translated regions are represented in the figure by the bold black line; untranslated regions are represented by the light black line.

The PaLoc location of the *tcdC* gene is shown in Fig. 2A and C. *tcdC* sequence data (552nt) from the initiation codon to codon 184 of the 233 amino acid protein were determined for all isolates. This sequence spans all previously described truncations and deletions within the dimerization domain [14], [33], [34]. A total of 26 different alleles were identified (Fig. 4). Each allele was assigned a number in the order of discovery and the sequences were made available at http://pubmlst.org/cdifficile. The 26 different *tcdC* variants included nine of 15 *tcdC* alleles described previously [34] and a further 17 alleles unique to the present study. The relationship between the *tcdC* alleles and the five clades identified in this population was examined using a neighbour joining tree. A mostly congruent association was demonstrated, the one exception being allele *tcdC*-12 which is found in ST-3 (clade 1), but has a sequence similar to the *tcdC* of clade 2 (Fig. 4A).

The *tcdC* variants were categorised and named as follows (Fig. 4B); (i) wild type (**WT**), (ii) modified by an 18 nucleotide deletion within the coiled-coil domain, (**Δ18**) and (iii) truncated, either by a single nucleotide deletion causing a frameshift and stop codon at position 66, (**Δ1stop**), or by a single nucleotide substitution creating a stop codon at position 62 (**TAAstop**).

All of the *tcdC* alleles in clades 1 and 4 isolates were wild type, lacking premature termination codons. However, two clade 1 alleles contained an 18 nucleotide dimerization domain deletion (Fig. 4). Both were derived from the most abundant *tcdC*-RBD allele, (**WT**tcdC-3), but had different 18 nucleotide deletions (Fig. 4, Table 1).

The nucleotide locations of all six Δ18 sequences identified (Fig. 4) were difficult to define precisely because they occur within repetitive sequences and more than one equally likely sequence alignment could be generated. However, ClustalW2 alignments showed that **Δ18**TcdC-25 was unique in having its Δ18 displaced by 9 nt relative to the other Δ18 containing alleles (alignment not shown).

Truncation of the TcdC protein relatively close to the N-terminus occurred in all clade 3 isolates, by the same nucleotide substitution (**TAAstop**) as seen in ST-11 (078), the only member of clade 5. This truncation was unique to clade 3 and ST-11 (078), which had closely related *tcdC* genes (Fig. 4A). Clade 3 and ST-11 (078) also had deletions within the untranslated nucleotide sequences of the coiled-coil domain (Fig. 4B); Δ18 and Δ54 in clade 3, and Δ36 in ST-11 (078). The similarity between ST-11 (078) and clade 3 within the two PaLoc loci sequenced (Fig. 3 and Fig. 4) was surprising, given the high divergence of the ST-11 (078) MLST loci from other known genotypes (Fig. 1). It raises the possibility that the ST-11 (078) PaLoc at least, was acquired by homologous recombination.

Clade 2 (containing ST-1 [027]), was the only clade to contain *tcdC* variants from all three categories; **WT**, **Δ18**, and **Δ1stop** (Fig. 4) and was therefore unusual in its heterogeneity with regard to this characteristic. However, all 448 ST-1 (027) isolates contained allele **Δ1stop** *tcdC*-1, encoding a truncated TcdC protein.

Almost all STs occurred with a single *tcdB*-RBD and *tcdC* variant; only a few had low frequency variants (Table 1). The two PaLoc loci did have higher dN/dS values than the housekeeping loci, but this was still much less than 1, and therefore not indicative of diversifying selection (Table S2). Overall, *tcdB*-RBD and *tcdC* variants were highly predictive of clade, and in clade 4 and clade 5 also predictive of ST.

## Discussion

The population structure of a large (n = 1290), recent collection of clinical *C. difficile* isolates, representing a population unit of circulating strains, was defined using MLST. The sequences of two loci (PaLoc *tcdB*-RBD and *tcdC*) putatively linked to hypervirulence [18], [24], [30], [31], [33], [34] were determined, mapped onto the population structure, and used to examine the underlying evolutionary mechanisms.

Our data confirm the clonal population structure of *C. difficile* [22], [35], [36] and demonstrate a largely congruent association between clade and PaLoc *tcdB*-RBD and *tcdC* variants. Only occasional deviations from congruence were identified due to recombination events. STs sharing the same PCR-ribotype were in most cases closely related (supported by bootstraps, Fig. S1 and Table S3), further supporting the clonal population structure. These observations are in agreement with previous suggestions based on nucleotide sequences [34], [36], [38], toxinotyping (a RFLP-PCR based method in which two PCR amplified fragments from the *tcdB* and *tcdA* genes undergo restriction digest to give characteristic banding patterns) and PCR-ribotyping [48], [49]. However, since MLST data allow the precise phylogenetic relationships among genotypes to be visualised (Fig. 1) the present study demonstrates that specific PaLoc variants (Fig. 4) are clade-associated. The ability to cluster genetically related isolates may provide greater power in future studies aiming to investigate associations between clinical disease severity and genotype.

The five clades defined by traditional phylogenetic approaches (Fig. 1A, 1B and [22]) were supported by ClonalFrame analysis (Fig. 1C, Fig. S1). ClonalFrame showed that recombination had an effect approximately four times lower than point mutation (r/m = 0.25 with credibility interval [0.12;0.42]). This is consistent with a previous estimate of r/m = 0.2, [50] also based on MLST data [35]. A significantly higher value of r/m between 0.63 and 1.13 has also been reported in the deep phylogeny of *C. difficile* based on whole genomes [37]. The authors suggested that this difference may reflect recombination rates that are lower in housekeeping genes than the genome as a whole.

Although clade 1 contained by far the highest number of STs, further work studying additional isolates from diverse sources may identify additional genotypes within the other clades. STs submitted by other laboratories to the MLST database (http://pubmlst.org/cdifficile) suggest this is the case, the exception currently being clade 5, containing only ST-11 (078). The high frequency and large number of different clade 1 genotypes (Table 1) implies that this clade may be particularly well adapted to humans, and therefore potentially sampled most frequently.

All isolates were cultured from ELISA positive stools (indicating the presence of toxin A and, or toxin B) and screened for the PaLoc by lok 1/3 PCR [47]. Eighteen non-toxigenic isolates were identified suggesting either simultaneous colonisation with a toxigenic strain, or an unreliable false positive ELISA test result, which may occur in as many as 20% of cases. All isolates that contained the Paloc genes *tcdB* and *tcdC* were negative for the lok1/3 PCR [47], indicating that despite the high mobility of the *C. difficile* genome [51], the PaLoc (in this clinical isolate population) remains in the same chromosomal location defined 14 years ago [47]. This, together with the observation that *tcdB*-RBD and *tcdC* sequences are largely congruent with clade, may indicate that the PaLoc inserted into the genome once, prior to the divergence of the clades. Subsequent homologous recombination may have imported the divergent *tcdB* sequences found in clade 2 from another Clostridial species possibly on more than one occasion. Consistent with this, the *tcdB* of *C. difficile* strain 8864 is divergent throughout its length (GenBank AJ011301; [52]) and is closely related to both the *tcdB*-RBD found in ST-1 (027), and the *tcdB* N-terminal catalytic domain of ST-37 (017, A-B+, clade 4) [53]. TcdB sequences are therefore either 8864-like or CD630-like (ST-54, clade 1, [51]), or mosaics of the two. An alternative explanation for the observed congruence of clade and PaLoc is that the PaLoc inserts in a nucleotide sequence and lineage specific manner, possibly in the form of a clade-specific bacteriophage. The latter is supported by the occurrence of non-toxigenic STs throughout clade 1, and in clade 4 (Fig. 1). Three STs had both toxigenic and non-toxigenic variants (Fig. 1). Interestingly, the eBURST diagram (Fig. 1B) [45] showed that the three STs (ST-3, ST-7, and ST-48) identified in both toxigenic and non-toxigenic form were single locus variants clustering closely together. This may indicate PaLoc instability within a common genetic background.

Putative hypervirulence features of the ST-1 (027) PaLoc (relating to increased toxin production) [18], [24], [30], [31], [33], [34] were not exclusive to this genotype. The divergent *tcdB*-RBD sequence occurred throughout clade 2 (Fig. 3) and two clade 2 ST-41 isolates had the same *tcdC* truncation as ST-1 (Fig. 4). However, the very low incidence of these isolates (Table 1) suggests they differ from ST-1 in transmissibility and/or other determinants of pathogenicity.

The *tcdB*-RBD and *tcdC* loci of clade 5 ST-11 (078) were closely related to clades 1 to 4 (Fig. 3 and Fig. 4), in contrast to its MLST loci which were divergent from the other clades (Fig. 1). Furthermore, the PaLoc *tcdC* of clade 3 and clade 5 ST-11 (078) uniquely shared the same nucleotide substitution that truncates the protein. This raises the possibility that clade 3 may, (like clade 5 hypervirulent ST-11 078) have high virulence potential, a hypothesis that will be tested using clinical severity data collected for these isolates. Clade 3 is associated with CDI, causing 49 cases during the study (3.8%), compared to 27 (2.1%) cases due to ST-11 (078). National surveillance data for England show that clade 3 associated PCR-ribotype 023 was endemic in the South during this study period [17], the incidence peaking at ∼18% (London region, April to June 2007). PCR-ribotype 023 represented 43 of 2030 (2.1%) UK isolates collected during the 1990s [54] and has been detected in Poland and Finland [55], [56].

Truncation of TcdC occurred by two different mechanisms; a single nucleotide deletion (in some clade 2 isolates) and a single nucleotide substitution (common to all members of clades 3 and 5) (Fig. 4). The evolution of this truncation at least twice may indicate evolutionary convergence due to a common selective advantage. These three clades are associated with clinically more severe disease relative to clades 1 and 4 (data not shown).

Three STs in clade 1 (ST-9, ST-10 and ST-51) are of interest as they occur with both wild type *tcdC*, and a coiled-coil domain Δ18 (Fig. 4, Table 1). This deletion did not impact on *tcdC* function in a *tcdA*-β-glucuronidase reporter fusion constructed in *C. perfringens* [14]. The naturally occurring paired Δ18*tcdC* and wild type *tcdC* variants we describe could be used to confirm these observations. Our mutants harboured the Δ18 nt in two different locations, suggesting the nucleotide repeats of the coiled-coil domain are unstable, with the 18 nt deletion arising more than once (Fig. 4).

We intend to test the hypothesis that specific STs and, or PaLoc variants are associated with more or less severe disease, using clinical data collected for this large cohort of CDI cases. Data on the relative pathogenicity of different genotypes would assist patient management, targeting of infection control resources and the identification of emergent hypervirulent strains. This large data set provides a framework for further study of *C. difficile* population biology, and establishes a baseline against which isolates from different hosts and geographic regions can be compared, to understand the sources and evolutionary origins of *C. difficile* strains that currently cause infection in humans.

## Materials and Methods

### Ethics Statement

This study focused only on characterising *C. difficile* isolates that were archived on an ongoing basis. As this study did not use any patient data, the research ethics committee advised that ethical approval was not required.

### Isolates

All sequential *C. difficile* positive stool samples identified by enzyme immunoassay (EIA) (Premier Toxins A&B Enzyme Immunoassay; Meridian Bioscience Europe, Italy) at the Clinical Microbiology Laboratory, Oxford Radcliffe Hospitals NHS Trust, Oxford, UK, between September 2006 and December 2009 were targeted for inclusion in this study. Approximately 70% of EIA positive stools from September 2006 to August 2007, and approximately 95% from September 2007 to December 2009 were retained, and contained sufficient faecal sample for culture, performed as in [22]. The routinely submitted faecal samples were obtained from both hospital and community patients. The size of the population served is approximately 600,000, which represents around 1% of the UK population. When more than one faecal sample received from a single patient yielded isolates of the same genotype, only the first isolate was included. A total of 1290 isolates were available for study, representing 1217 patients and 1277 episodes of diarrhoea (based on a 14 day de-duplication).

### Genotyping

MLST and PCR-ribotyping were performed as described previously [22]. The composition of all PCRs was as before, [22] with additional oligonucleotide primers as follows. Absence of the PaLoc was confirmed using PCR primer pair lok1 and lok3 [22], [42] (Fig. 2). The *tcdB*-RBD fragment was amplified and sequenced using oligonucleotide primer pair tcdB3 5′-GTAGTTGGATGGAARGATTTAG-3′ and tcdB4 5′-CATCYAAAGTATTTTGATGTGC-3′ (712bp amplicon). Amplification conditions were 95°C for 15s, followed by 35 cycles of 94°C for 30 s, 50°C for 40 s, and 72°C for 1 min 10 s, then 72°C for 5 min. The *tcdC* sequence was amplified and sequenced using primer pair tcdC-F1 5′ AATTTTTAGTCAACTAGTTATTTTAAG-3′ (located 75 nt upstream of the *tcdC* initiation codon) tcdC-R1 5′-TATAGTTCCAGCACTTATACCTC-3′ (688 bp amplicon). Amplification conditions were 95°C for 15s, followed by 35 cycles of 94°C for 30 s, 59°C for 30 s, and 72°C for 1 min, then 72°C for 5 min. High throughput nucleotide sequencing was performed as described [22]. MLST or *tcdB*-RBD and *tcdC* sequencing of all the isolates giving a newly identified allelic profile (ST) or allele nucleotide sequence was performed at least twice, each time using newly extracted DNA from the isolate to confirm the result.

### Phylogenetic Analysis

Manual alignments of nucleotide sequences containing deletions were prepared using BioEdit Sequence Alignment Editor [57], and using the program ClustalW2 (http://www.ebi.ac.uk/Tools/clustalw2/index.html). Neighbour joining trees were constructed using MEGA version 4 (available from http://www.megasoftware.net/) [58]. Phylogenetic networks were constructed using Neighbour-Net (part of the SplitsTree4 software package, http://www.splitstree.org) [59]. eBURST [45] was used to investigate relationship among STs on the basis of alleleic profiles. ClonalFrame analysis [46] was performed by preparing an extended multi-FASTA file containing one representative of each of the 78 STs. ClonalFrame reconstructs genealogies in a similar fashion to traditional phylogenetic techniques, with the difference that it detects, quantifies and accounts for the effect of homologous recombination. ClonalFrame was run for 100,000 iterations, the first half of which was discarded to allow for convergence. Convergence and mixing were found to be suitable by comparison of four independent runs.

## Supporting Information

Figure S1 **Clonal population structure is supported by clustering of STs sharing the same ribotype.** ClonalFrame analysis of all 78 STs as shown in Fig. 1C. PCR-ribotypes which occurred with more than one ST (Table S3) are mapped onto the tree and given a unique colour.
(TIF)

Table S1 **Frequency of different STs within the clinical isolate dataset (n = 1290), ranked in descending order of abundance.** The clade of each ST is indicated, followed by the number of non-toxigenic isolates of each ST, and the frequency of the different *tcdB* and *tcdC* alleles associated with toxigenic variants of each ST. N/A: not applicable as all isolates of this ST were non-toxigenic.
(DOC)

Table S2 **dN/dS values for MLST and PaLoc loci.**
(XLS)

Table S3 **Association of PCR-ribotype and ST.** A total of 285 isolates were PCR-ribotyped; 242 isolates during the study and 43 isolates previously [22]. STs occurring with more than one PCR-ribotype and PCR-ribotypes occurring with more than one ST are shown in the table. The following PCR-ribotypes occurred with one ST (>1 isolate typed): 027-ST1 (n = 46), 002-ST8 (n = 12), 078-ST11 (n = 6), 018-ST17 (n = 6), 026-ST7 (n = 4), 081-ST9 (n = 4), 137-ST4 (n = 4), 017-ST37 (n = 3), 012-ST54 (n = 2). The following PCR-ribotypes occurred with one ST (1 isolate typed): 070-ST55, 129-ST13, 046-ST35, 216-ST33, 320-ST46, 010-ST15, 053-ST63, 054-ST43, 022-ST66, 316-ST59, 062-ST75, 097-ST21, 019-ST67, 319-ST74, 139-ST52, 110-ST19, 202-ST24, 140-ST26, 228-ST92, 326-ST91, 323-ST31, 138-ST23.
(XLSX)

## References

[1] Karas JA, Enoch DA, Aliyu SH (2010). A review of mortality due to *Clostridium difficile* infection.. J Infect.

[2] Rupnik M, Wilcox MH, Gerding DN (2009). *Clostridium difficile* infection: new developments in epidemiology and pathogenesis.. Nature Reviews Microbiology.

[3] Bartlett JG (2002). Clinical practice: antibiotic-associated diarrhea.. N Engl J Med.

[4] Chang JY, Antonopoulos DA, Kalra A, Tonelli A, Khalife WT (2008). Decreased diversity of the fecal Microbiome in recurrent *Clostridium difficile*-associated diarrhea.. J Infect Dis.

[5] Miller M, Gravel D, Mulvey M, Taylor G, Boyd D (2010). Health care-associated *Clostridium difficile* infection in Canada: patient age and infecting strain type are highly predictive of severe outcome and mortality.. Clin Infect Dis.

[6] von Eichel-Streiber C, Laufenberg-Feldmann R, Sartingen S, Schulze J, Sauerborn M (1992). Comparative sequence analysis of the *Clostridium difficile* toxins A and B. Mol Gen Genet.

[7] Albesa-Jové D, Bertrand T, Carpenter EP, Swain GV, Lim J (2010). Four distinct structural domains in *Clostridium difficile* toxin B visualized using SAXS.. J Mol Biol.

[8] Pruitt RN, Chambers MG, Ng KK, Ohi MD, Lacy DB (2010). Structural organization of the functional domains of *Clostridium difficile* toxins A and B. Proc Natl Acad Sci U S A.

[9] Lyras D, O'Connor JR, Howarth PM, Sambol SP, Carter GP (2009). Toxin B is essential for virulence of *Clostridium difficile*.. Nature.

[10] Kuehne SA, Cartman ST, Heap JT, Kelly ML, Cockayne A (2010). The role of toxin A and toxin B in *Clostridium difficile* infection.. Nature.

[11] Hundsberger T, Braun V, Weidmann M, Leukel P, Sauerborn M (1997). Transcription analysis of the genes *tcdA*-*E* of the pathogenicity locus of *Clostridium difficile*.. Eur J Biochem.

[12] Hammond GA, Lyerly DM, Johnson JL (1997). Transcriptional analysis of the toxigenic element of *Clostridium difficile*.. Microb Pathog.

[13] Govind R, Vediyappan G, Rolfe RD, Fralick, JA (2006). Evidence that *Clostridium difficile* TcdC is a membrane-associated protein.. J Bacteriol.

[14] Matamouros S, England P, Dupuy B (2007). *Clostridium difficile* toxin expression is inhibited by the novel regulator TcdC.. Mol Microbiol.

[15] Clabots CR, Johnson S, Bettin KM, Mathie PA, Mulligan ME (1993). Development of a rapid and efficient restriction endonuclease analysis typing system for *Clostridium difficile* and correlation with other typing systems.. J Clin Microbiol.

[16] Killgore G, Thompson A, Johnson S, Brazier J, Kuijper E (2006). Comparison of seven techniques for typing international epidemic strains of *Clostridium difficile*: restriction endonuclease analysis, pulsed-field gel electrophoresis, PCR-ribotyping, multilocus sequence typing, multilocus variable-number tandem-repeat analysis, amplified fragment length polymorphism, and surface layer protein A gene sequence typing.. J Clin Microbiol.

[17] Health Protection Agency (2009). *Clostridium difficile* Ribotyping Network for England and Northern Ireland: 2008/09 report. Health Protection Agency, London, United Kingdom.. http://www.hpa.org.uk/web/HPAwebFile/HPAweb_C/1258560554236.

[18] McDonald LC, Killgore GE, Thompson A, Owens RC, Kazakova SV (2005). An epidemic, toxin gene-variant strain of *Clostridium difficile*.. N Engl J Med.

[19] Pépin J, Valiquette L, Cossette B (2005). Mortality attributable to nosocomial *Clostridium difficile*-associated disease during an epidemic caused by a hypervirulent strain in Quebec.. CMAJ.

[20] Kuijper EJ, van den Berg RJ, Debast S, Visser CE, Veenendaal D (2006). *Clostridium difficile* ribotype 027, toxinotype III, the Netherlands.. Emerg Infect Dis.

[21] Health Protection Agency (2005). Outbreak of *Clostridium difficile* infection in a hospital in South East England. Commun Dis Rep CDR Wkly 15:2-3.. http://www.hpa.org.uk/cdr/archives/2005/cdr2405.pdf.

[22] Griffiths D, Fawley W, Kachrimanidou M, Bowden R, Crook DW (2010). Multilocus sequence typing of *Clostridium difficile*.. J Clin Microbiol.

[23] Merrigan M, Venugopal A, Mallozzi M, Roxas B, Viswanathan VK (2010). Human hypervirulent *Clostridium difficile* strains exhibit increased sporulation as well as robust toxin production.. J Bacteriol.

[24] Warny M, Pepin J, Fang A, Killgore G, Thompson A (2005). Toxin production by an emerging strain of *Clostridium difficile* associated with outbreaks of severe disease in North America and Europe.. Lancet.

[25] Freeman J, Baines SD, Saxton K, Wilcox MH (2007). Effect of metronidazole on growth and toxin production by epidemic *Clostridium difficile* PCR ribotypes 001 and 027 in a human gut model.. J Antimicrob Chemother.

[26] Akerlund T, Persson I, Unemo M, Noren T, Svenungsson B (2008). Increased Sporulation Rate of Epidemic *Clostridium difficile* Type 027/NAP1.. J Clin Microbiol.

[27] Goorhuis A, Bakker D, Corver J, Debast SB, Harmanus C (2008). Emergence of *Clostridium difficile* infection due to a new hypervirulent strain, polymerase chain reaction ribotype 078.. Clin Infect Dis.

[28] Jhung MA, Thompson AD, Killgore GE, Zukowski WE, Songer G (2008). Toxinotype V *Clostridium difficile* in humans and food animals.. Emerg Infect Dis.

[29] Burns K, Morris-Downes M, Fawley WN, Smyth E, Wilcox MH (2010). Infection due to *C. difficile* ribotype 078: first report of cases in the Republic of Ireland.. J Hosp Infect.

[30] Stabler RA, Gerding DN, Songer JG, Drudy D, Brazier JS (2006). Comparative phylogenomics of *Clostridium difficile* reveals clade specificity and microevolution of hypervirulent strains.. J Bacteriol.

[31] Stabler RA, Dawson LF, Phua LT, Wren BW (2008). Comparative analysis of BI/NAP1/027 hypervirulent strains reveals novel toxin B-encoding gene (*tcdB*) sequences.. J Med Microbiol.

[32] Lanis JM, Barua S, Ballard JD (2010). Variations in TcdB activity and the hypervirulence of emerging strains of *Clostridium difficile*.. PLoS Pathog.

[33] Spigaglia P, Mastrantonio P (2002). Molecular analysis of the pathogenicity locus and polymorphism in the putative negative regulator of toxin production (TcdC) among *Clostridium difficile* clinical isolates.. J Clin Microbiol.

[34] Curry SR, Marsh JW, Muto CA, O'Leary MM, Pasculle AW (2007). *tcdC* genotypes associated with severe TcdC truncation in an epidemic clone and other strains of *Clostridium difficile*.. J Clin Microbiol.

[35] Lemée L, Dhalluin A, Pestel-Caron M, Lemeland JF, Pons JL (2004). Multilocus sequence typing analysis of human and animal *Clostridium difficile* isolates of various toxigenic types.. J Clin Microbiol.

[36] Lemée L, Bourgeois I, Ruffin E, Collignon A, Lemeland JF (2005). Multilocus sequence analysis and comparative evolution of virulence-associated genes and housekeeping genes of *Clostridium difficile*.. Microbiology.

[37] He M, Sebaihia M, Lawley TD, Stabler RA, Dawson LF (2010). Evolutionary dynamics of *Clostridium difficile* over short and long time scales.. Proc Natl Acad Sci U S A.

[38] Marsh JW, O'Leary MM, Shutt KA, Sambol SP, Johnson S (2010). Multilocus variable-number tandem-repeat analysis and multilocus sequence typing reveal genetic relationships among *Clostridium difficile* isolates genotyped by restriction endonuclease analysis.. J Clin Microbiol.

[39] Hensgens MP, Goorhuis A, Notermans DW, van Benthem BH, Kuijper EJ (2009). Decrease of hypervirulent *Clostridium difficile* PCR ribotype 027 in the Netherlands.. Euro Surveill.

[40] Wilson V, Cheek L, Satta G, Walker-Bone K, Cubbon M (2010). Predictors of death after *Clostridium difficile* infection: a report on 128 strain-typed cases from a teaching hospital in the United Kingdom.. Clin Infect Dis.

[41] Gould LH, Limbago B (2010). *Clostridium difficile* in food and domestic animals: a new foodborne pathogen?. Clin Infect Dis.

[42] Belmares J, Johnson S, Parada JP, Olson MM, Clabots CR (2009). Molecular epidemiology of *Clostridium difficile* over the course of 10 years in a tertiary care hospital.. Clin Infect Dis.

[43] Clements AC, Magalhães RJ, Tatem AJ, Paterson DL, Riley TV (2010). *Clostridium difficile* PCR ribotype 027: assessing the risks of further worldwide spread.. Lancet Infect Dis.

[44] Bryant D, Moulton, V (2004). Neighbor-Net: An Agglomerative Method for the Construction of Phylogenetic Networks.. Mol Biol Evol.

[45] Feil EJ, Li BC, Aanensen DM, Hanage WP, Spratt BG (2004). eBURST: Inferring patterns of evolutionary descent among clusters of related bacterial genotypes from multilocus sequence typing data.. J Bact.

[46] Didelot X, Falush D (2007). Inference of bacterial microevolution using multilocus sequence data.. Genetics.

[47] Braun V, Hundsberger T, Leukel P, Sauerborn M, von Eichel-Streiber C (1996). Definition of the single integration site of the pathogenicity locus in *Clostridium difficile*.. Gene.

[48] Rupnik M, Avesani V, Janc M, von Eichel-Streiber C, Delmée M (1998). A novel toxinotyping scheme and correlation of toxinotypes with serogroups of *Clostridium difficile* isolates.. J Clin Microbiol.

[49] Rupnik M, Brazier JS, Duerden BI, Grabnar M, Stubbs SL (2001). Comparison of toxinotyping and PCR ribotyping of *Clostridium difficile* strains and description of novel toxinotypes.. Microbiology.

[50] Vos M, Didelot X (2009). A comparison of homologous recombination rates in bacteria and archaea.. ISME J.

[51] Sebaihia M, Wren BW, Mullany P, Fairweather NF, Minton N (2006). The multidrug-resistant human pathogen *Clostridium difficile* has a highly mobile, mosaic genome.. Nat Genet.

[52] Soehn F, Wagenknecht-Wiesner A, Leukel P, Kohl M, Weidmann M (1998). Genetic rearrangements in the pathogenicity locus of *Clostridium difficile* strain 8864; implications for transcription, expression and enzymatic activity of toxins A and B. Mol Gen Genet.

[53] Drudy D, Fanning S, Kyne L (2007). Toxin A-negative, toxin B-positive *Clostridium difficile*.. Int J Infect Dis.

[54] Stubbs SL, Brazier JS, O'Neill GL, Duerden BI (1999). PCR targeted to the 16S-23S rRNA gene intergenic spacer region of *Clostridium difficile* and construction of a library consisting of 116 different PCR ribotypes.. J Clin Microbiol.

[55] Pituch H, Brazier JS, Obuch-Woszczatynski P, Wultanska D, Meisel-Mikołajczyk F (2006). Prevalence and association of PCR ribotypes of *Clostridium difficile* isolated from symptomatic patients from Warsaw with macrolide-lincosamidestreptogramin B (MLSB) type resistance.. Journal of Medical Microbiology.

[56] Pasanen T, Kotila SM, Horsma J, Virolainen A, Jalava J (2011). Comparison of repetitive extragenic palindromic sequence-based PCR with PCR ribotyping and pulsed-field gel electrophoresis in studying the clonality of *Clostridium difficile*.. Clin Microbiol Infect.

[57] Hall TA (1999). BioEdit: a user-friendly biological sequence alignment editor and analysis program for Windows 95/98/NT.. Nucl Acids Symp Ser.

[58] Tamura K, Dudley J, Nei M, Kumar S (2007). MEGA4: Molecular Evolutionary Genetics Analysis (MEGA) software version 4.0.. Molecular Biology and Evolution.

[59] Huson DH, Bryant D (2006). Application of Phylogenetic Networks in Evolutionary Studies, Molecular Biology and Evolution.

[60] Dingle T, Wee S, Mulvey GL, Greco A, Kitova EN (2008). Functional properties of the carboxy-terminal host cell-binding domains of the two toxins, TcdA and TcdB, expressed by *Clostridium difficile*.. Glycobiology.

[61] Jolley KA, Maiden MCJ (2010). BIGSdb: Scalable analysis of bacterial genome variation at the population level.. BMC Bioinformatics.

