# Supplementary figures and images for: Clinical Clostridium difficile: Clonality and Pathogenicity Locus Diversity
Source: PLoS One. 2011 May 19;6(5):e19993. doi: 10.1371/journal.pone.0019993 (PMC3098275; doi:10.1371/journal.pone.0019993)

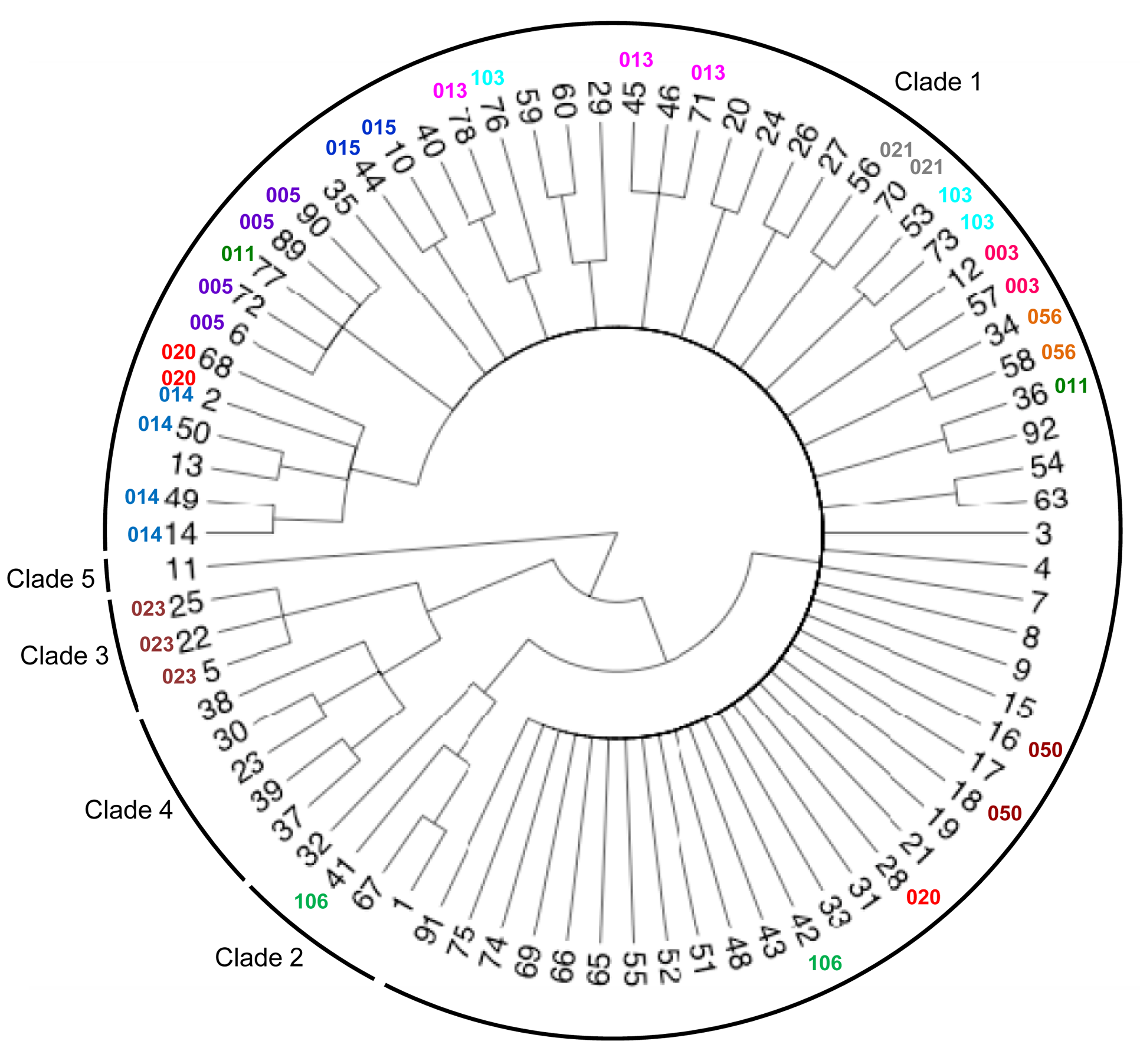

Supplement: Figure S1 — Clonal population structure is supported by clustering of STs sharing the same ribotype. ClonalFrame analysis of all 78 STs as shown in Fig. 1C. PCR-ribotypes which occurred with more than one ST (Table S3) are mapped onto the tree and given a unique colour. (TIF) [file pone.0019993.s001.tif]
