# Supplementary material for: Clinical Clostridium difficile: Clonality and Pathogenicity Locus Diversity
Source: PLoS One. 2011 May 19;6(5):e19993. doi: 10.1371/journal.pone.0019993 (PMC3098275; doi:10.1371/journal.pone.0019993)
Supplement: Table S1 — Frequency of different STs within the clinical isolate dataset (n = 1290), ranked in descending order of abundance. The clade of each ST is indicated, followed by the number of non-toxigenic isolates of each ST, and the frequency of the different tcdB and tcdC alleles associated with toxigenic variants of each ST. N/A: not applicable as all isolates of this ST were non-toxigenic. (DOC) [file pone.0019993.s002.doc]

**TABLE S1**

| **ST (n)** | **Clade** | **Non-toxigenic (n)** | ***tcdB*-RBD allele (n)** | ***tcdC* allele (n)** |
| --- | --- | --- | --- | --- |
| **1** (448) | 2 | 0 | tcdB-RBD8 (448) | D1stop tcdC-1 (448) |
| **2** (86) | 1 | 0 | tcdB-RBD3 (86) | WT tcdC-4 (85) |
|  |  |  |  | WT tcdC-3 (1) |
| **8** (86) | 1 | 0 | tcdB-RBD3 (86) | WT tcdC-3 (86) |
| **42** (68) | 1 | 0 | tcdB-RBD3 (67) | WT tcdC-3 (68) |
|  |  |  | tcdB-RBD11 (1) | |
| **6** (59) | 1 | 0 | tcdB-RBD3 (58) | WT tcdC-2 (59) |
|  |  |  | tcdB-RBD14 (1) | |
| **3** (54) | 1 | 2 | tcdB-RBD3 (52) | WT tcdC-2 (50) |
|  |  |  |  | WT tcdC-3 (1) |
|  |  |  |  | WT tcdC-12 (1) |
| **44** (46) | 1 | 0 | tcdB-RBD3 (45) | WT tcdC-3 (46) |
|  |  |  | tcdB-RBD11 (1) | |
| **5** (43) | 3 | 0 | tcdB-RBD5 (43) | TAAstop tcdC-9 (39) |
|  |  |  |  | TAAstop tcdC-20 (4) |
| **10** (43) | 1 | 0 | tcdB-RBD3 (43) | D18 tcdC-7 (34) |
|  |  |  |  | WT tcdC-3 (9) |
| **14** (28) | 1 | 0 | tcdB-RBD3 (28) | WT tcdC-4 (28) |
| **11** (27) | 5 | 0 | tcdB-RBD1 (27) | TAAstop tcdC-5 (27) |
| **9** (22) | 1 | 0 | tcdB-RBD3 (22) | WT tcdC-3 (20) |
|  |  |  |  | D18 tcdC-25 (2) |
| **7** (20) | 1 | 7 | tcdB-RBD3 (13) | WT tcdC-2 (12) |
|  |  |  |  | WT tcdC-16 (1) |
| **37** (19) | 4 | 0 | tcdB-RBD9 (19) | WT tcdC-6 (19) |
| **17** (18) | 1 | 0 | tcdB-RBD3 (18) | WT tcdC-8 (16) |
|  |  |  |  | WT tcdC-3 (1) |
|  |  |  |  | WT tcdC-11 (1) |
| **58** (18) | 1 | 0 | tcdB-RBD7 (14) | WT tcdC-10 (14) |
|  |  |  | tcdB-RBD4 (4) | WT tcdC-8 (4) |
| **49** (17) | 1 | 0 | tcdB-RBD3 (17) | WT tcdC-4 (17) |
| **16** (14) | 1 | 0 | tcdB-RBD3 (14) | WT tcdC-3 (14) |
| **13** (12) | 1 | 0 | tcdB-RBD3 (12) | WT tcdC-4 (12) |
| **54** (12) | 1 | 0 | tcdB-RBD2 (12) | WT tcdC-4 (12) |
| **33** (11) | 1 | 0 | tcdB-RBD2 (11) | WT tcdC-14 (11) |
| **36** (11) | 1 | 0 | tcdB-RBD3 (11) | WT tcdC-8 (11) |
| **45** (11) | 1 | 0 | tcdB-RBD3 (11) | WT tcdC-3 (11) |
| **18** (10) | 1 | 0 | tcdB-RBD3 (10) | WT tcdC-3 (10) |
| **12** (8) | 1 | 0 | tcdB-RBD3 (8) | WT tcdC-3 (7) |
|  |  |  |  | WT tcdC-23 (1) |
| **55** (8) | 1 | 0 | tcdB-RBD3 (8) | WT tcdC-4 (8) |
| **35** (7) | 1 | 0 | tcdB-RBD6 (7) | WT tcdC-3 (7) |
|  |  |  | tcdB-RBD3 (1) |  |
| **53** (7) | 1 | 0 | tcdB-RBD3 (7) | WT tcdC-21 (6) |
|  |  |  |  | WT tcdC-22 (1) |
| **15** (6) | 1 | 6 | N/A | N/A |
| **43** (6) | 1 | 0 | tcdB-RBD3 (6) | WT tcdC-3 (6) |
| **22** (5) | 3 | 0 | tcdB-RBD5 (5) | TAAstop tcdC-9 (5) |
| **63** (5) | 1 | 0 | tcdB-RBD3 (5) | WT tcdC-3 (5) |
| **46** (4) | 1 | 0 | tcdB-RBD3 (4) | WT tcdC-3 (4) |
| **4** (3) | 1 | 0 | tcdB-RBD2 (3) | WT tcdC-3 (3) |
| **41** (3) | 2 | 0 | tcdB-RBD10 (2) | D18 tcdC-24 (1) |
|  |  |  | tcdB-RBD8 (1) | D1stop tcdC-15 (1) |
|  |  |  |  | D1stop tcdC-26 (1) |
| **48** (3) | 1 | 1 | tcdB-RBD17 (1) | WT tcdC-3 (1) |
|  |  |  | tcdB-RBD3 (1) | WT tcdC-8 (1) |
| **56** (3) | 1 | 0 | tcdB-RBD3 (3) | WT tcdC-8 (3) |
| **72** (3) | 1 | 0 | tcdB-RBD3 (3) | WT tcdC-2 (3) |
| **28** (2) | 1 | 0 | tcdB-RBD3 (2) | WT tcdC-4 (1) |
|  |  |  |  | WT tcdC-2 (1) |
| **51** (2) | 1 | 0 | tcdB-RBD3 (2) | D18 tcdC-7 (1) |
|  |  |  |  | WT tcdC-3 (1) |
| **57** (2) | 1 | 0 | tcdB-RBD3 (2) | WT tcdC-3 (2) |
| **75** (2) | 1 | 0 | tcdB-RBD2 (2) | WT tcdC-17 (1) |
|  |  |  |  | WT tcdC-3 (1) |
| **77** (2) | 1 | 0 | tcdB-RBD3 (2) | WT tcdC-8 (2) |
| **19** (1) | 1 | 0 | tcdB-RBD3 (1) | WT tcdC-3 (1) |
| **21** (1) | 1 | 0 | tcdB-RBD11 (1) | WT tcdC-3 (1) |
| **23** (1) | 4 | 1 | N/A | N/A |
| **24** (1) | 1 | 0 | tcdB-RBD3 (1) | WT tcdC-3 (1) |
| **25** (1) | 3 | 0 | tcdB-RBD5 (1) | TAAstop tcdC-9 (1) |
| **26** (1) | 1 | 1 | N/A | N/A |
| **31** (1) | 1 | 0 | tcdB-RBD3 (1) | WT tcdC-2 (1) |
| **34** (1) | 1 | 0 | tcdB-RBD4 (1) | WT tcdC-3 (1) |
| **50** (1) | 1 | 0 | tcdB-RBD3 (1) | WT tcdC-4 (1) |
| **52** (1) | 1 | 0 | tcdB-RBD3 (1) | WT tcdC-4 (1) |
| **59** (1) | 1 | 0 | tcdB-RBD12 (1) | WT tcdC-13 (1) |
| **60** (1) | 1 | 0 | tcdB-RBD3 (1) | WT tcdC-3 (1) |
| **65** (1) | 1 | 0 | tcdB-RBD3 (1) | WT tcdC-18 (1) |
| **66** (1) | 1 | 0 | tcdB-RBD3 (1) | WT tcdC-3 (1) |
| **67** (1) | 2 | 0 | tcdB-RBD13 (1) | WT tcdC-19 (1) |
| **68** (1) | 1 | 0 | tcdB-RBD3 (1) | WT tcdC-4 (1) |
| **70** (1) | 1 | 0 | tcdB-RBD3 (1) | WT tcdC-8 (1) |
| **71** (1) | 1 | 0 | tcdB-RBD3 (1) | WT tcdC-3 (1) |
| **73** (1) | 1 | 0 | tcdB-RBD3 (1) | WT tcdC-3 (1) |
| **74** (1) | 1 | 0 | tcdB-RBD3 (1) | WT tcdC-8 (1) |
| **76** (1) | 1 | 0 | tcdB-RBD3 (1) | WT tcdC-3 (1) |
| **78** (1) | 1 | 0 | tcdB-RBD3 (1) | WT tcdC-3 (1) |
| **89** (1) | 1 | 0 | tcdB-RBD3 (1) | WT tcdC-2 (1) |
| **90** (1) | 1 | 0 | tcdB-RBD3 (1) | WT tcdC-3 (1) |
| **91** (1) | 1 | 0 | tcdB-RBD3 (1) | WT tcdC-3 (1) |
| **92** (1) | 1 | 0 | tcdB-RBD3 (1) | WT tcdC-22 (1) |
